# Glial Glutamate Transporter Modulation Prevents Development of Complete Freund’s Adjuvant-Induced Hyperalgesia and Allodynia in Mice

**DOI:** 10.3390/brainsci13050807

**Published:** 2023-05-16

**Authors:** Ghallab Alotaibi, Amna Khan, Patrick J. Ronan, Kabirullah Lutfy, Shafiqur Rahman

**Affiliations:** 1 Department of Pharmaceutical Sciences, College of Pharmacy, South Dakota State University, Brookings, SD 57007, USA; 2 Research Service, Sioux Falls VA Healthcare System, Sioux Falls, SD 57105, USA; 3 Department of Psychiatry and Basic Biomedical Sciences, University of South Dakota Sanford School of Medicine, Sioux Falls, SD 57105, USA; 4 College of Pharmacy, Western University of Health Sciences, Pomona, CA 91766, USA

**Keywords:** glutamate transporter, chronic pain, astroglia, microglia, LDN-212320, mice

## Abstract

Glial glutamate transporter (GLT-1) modulation in the hippocampus and anterior cingulate cortex (ACC) is critically involved in nociceptive pain. The objective of the study was to investigate the effects of 3-[[(2-methylphenyl) methyl] thio]-6-(2-pyridinyl)-pyridazine (LDN-212320), a GLT-1 activator, against microglial activation induced by complete Freund’s adjuvant (CFA) in a mouse model of inflammatory pain. Furthermore, the effects of LDN-212320 on the protein expression of glial markers, such as ionized calcium-binding adaptor molecule 1 (Iba1), cluster of differentiation molecule 11b (CD11b), mitogen-activated protein kinases (p38), astroglial GLT-1, and connexin 43 (CX43), were measured in the hippocampus and ACC following CFA injection using the Western blot analysis and immunofluorescence assay. The effects of LDN-212320 on the pro-inflammatory cytokine interleukin-1β (IL-1β) in the hippocampus and ACC were also assessed using an enzyme-linked immunosorbent assay. Pretreatment with LDN-212320 (20 mg/kg) significantly reduced the CFA-induced tactile allodynia and thermal hyperalgesia. The anti-hyperalgesic and anti-allodynic effects of LDN-212320 were reversed by the GLT-1 antagonist DHK (10 mg/kg). Pretreatment with LDN-212320 significantly reduced CFA-induced microglial Iba1, CD11b, and p38 expression in the hippocampus and ACC. LDN-212320 markedly modulated astroglial GLT-1, CX43, and, IL-1β expression in the hippocampus and ACC. Overall, these results suggest that LDN-212320 prevents CFA-induced allodynia and hyperalgesia by upregulating astroglial GLT-1 and CX43 expression and decreasing microglial activation in the hippocampus and ACC. Therefore, LDN-212320 could be developed as a novel therapeutic drug candidate for chronic inflammatory pain.

## 1. Introduction

Chronic inflammatory pain is a devastating condition causing enormous physical and emotional burden on millions of people worldwide [1]. “Current analgesics are less effective in relieving chronic pain symptoms and often associated with severe adverse effects including numerous central nervous system related complications, gastrointestinal and renal side effects” [2,3]. Furthermore, the current pain medications have inadequate efficacy due to the limited availability to the site of action [2]. Thus, there is a need to develop effective medications to treat chronic inflammatory pain by targeting novel targets and mechanisms in the central nervous system (CNS).

Emerging evidence indicates that the activation of microglial cells and subsequent release of proalgesic mediators play a critical role in chronic pain facilitation following peripheral tissue injury, such as complete Freud’s adjuvant (CFA)-induced chronic inflammation [4]. Moreover, previous studies have shown that inhibiting microglial activation reduces hyperalgesia (i.e., increased pain sensitivity to noxious stimuli) and allodynia (i.e., pain sensation to non-noxious stimuli), common symptoms of chronic inflammatory pain [5,6,7,8,9]. A growing body of evidence has indicated that microglial activation causes the upregulation of markers and production of proinflammatory mediators [8]. For example, microglial activation accompanies increased expression of ionized calcium-binding adaptor molecule 1 (Iba1) and cluster of differentiation molecule 11b (CD11b) in the CNS following tissue injury [10]. Furthermore, the microglial production of cytokines including interleukin-1β (IL-1β) promotes the development of allodynia following inflammatory pain insults [11,12]. Additional reports have indicated that microglial activation increases the release of mitogen-activated protein kinase (p38) in the CNS in inflammatory pain models [13].

The accumulated evidence suggests that the hippocampus and anterior cingulate cortex (ACC) are critically involved in central pain processing [14,15,16]. For example, the enhancement of glutamate ionotropic receptor-mediated functions in the ACC contributes to increased pain symptoms such as allodynia in animal models of inflammatory pain [17,18,19]. Furthermore, previous findings showed that glutamate receptor expression was altered following peripheral nerve injury in the hippocampus [20]. It is noteworthy to mention that the neurotransmitter glutamate plays a critical role in the CNS in the transmission of chronic pain [21,22]. Moreover, increased glutamate release is considered an important central mechanism underlying the enhancement of neuronal responsiveness in the hippocampus and ACC in neuropathic pain [20,23,24] or inflammatory pain induced by CFA [25] or formalin [26]. For instance, previous studies have shown that glutamatergic neurotransmission in the CNS contributes to the development and maintenance of allodynia and hyperalgesia in peripheral tissue injury [27,28]. Notably, increased glutamate neurotransmission facilitates the activation of microglial-induced mediator release following peripheral nerve injury in the CNS through glutamate ionotropic receptors [27].

It is widely recognized that the glutamate concentration is regulated by glutamate transporters in the CNS [29,30]. Astroglial glutamate transporter (GLT-1), or the human homologue excitatory amino acid transporter (EAAT2), plays a crucial role in the modulation of synaptic transmission through the regulation of extracellular glutamate levels [31,32,33]. The astroglial GLT-1 is a major glutamate transporter responsible for about 90% of the total glutamate uptake in the CNS, with prominent expression in the hippocampus and cortex [32,34,35,36]. Furthermore, previous studies have shown that astroglial GLT-1 modulation produced analgesia in an animal model of nociceptive pain [26,37]. Another study showed that gabapentin attenuated neuropathic pain through a GLT-1-dependent mechanism [38]. Furthermore, the expression levels of astroglial GLT-1 and connexin 43 (CX43) are both significantly reduced following peripheral inflammation [39,40]. Importantly, decreased gabapentin activity is associated with decreased astroglial GLT-1 expression [41]. Overall, these studies show that enhanced astroglial GLT-1 activity provides anti-nociceptive properties in various pain models. However, the role of astroglial GLT-1 modulation in CFA-induced microglial activation-associated hyperalgesia and allodynia remains unknown. In the present study, we have investigated the effects of LDN-212320, a GLT-1 activator, on CFA-induced hyperalgesia and allodynia. We also examined the effects of LDN-212320 on microglial activation by measuring the expression of microglial markers such as Iba1, CD11b, p38, astroglial GLT-1, and CX43 in the hippocampus and ACC using several biochemical and molecular techniques.

## 2. Materials and Methods

### 2.1. Animals

Male C57BL/6J mice (weighing 20–30 g; 7–9 weeks old) were purchased from Jackson laboratories (Bar Harbor, ME, USA). The animals were allowed to acclimatize for 7 days prior to any behavioral experiments. The mice were housed in standard cages (29 × 18 × 12 cm) with free access to standard laboratory chow and water, under standard laboratory conditions (22 ± 2 °C, relative humidity 60%), and maintained on a regular 12 h light/dark cycle (lights on at 0700 h). All behavioral experiments were conducted between regular light cycles (09:00–17:00 h). On the day of the behavioral experiments, the mice were allowed to habituate to the testing room for at least 30 min before starting the experiments. All procedures used in this study followed the National Institutes of Health guidelines for the Care and Use of Laboratory Animals and were approved by the Institutional Animal Care and Use Committee at South Dakota State University under approval number 19-040A. The Good Laboratory Practice and ARRIVE guidelines were followed. All efforts were made to ensure minimal animal suffering.

### 2.2. Drugs and Treatment

LDN-212320 (Axon Medchem, Reston, VA, USA) was dissolved in normal saline (0.9% NaCl) with 1% dimethyl sulphoxide (DMSO) and 0.5% Tween-80 (vehicle). The solution composition for the LDN was selected based on the reported solubility and stability of this compound. We have previously reported this composition elsewhere [26].

**Figure 1:**
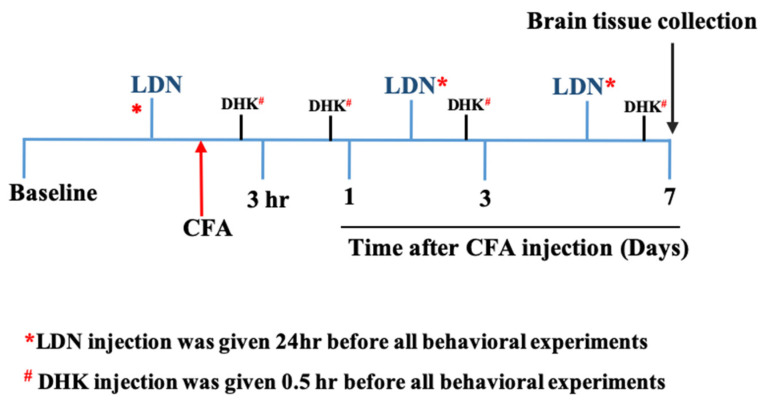
The diagram depicts the timeline of the experiments. CFA, complete Freund’s adjuvant; LDN, 3-[[(2-methylphenyl) methyl] thio]-6-(2-pyridinyl)-pyridazine; DHK, dihydrokainic acid.

Dihydrokainic acid (DHK) and gabapentin (positive control), purchased from Sigma-Aldrich (St. Louis, MO, USA), were dissolved in normal saline (0.9% NaCl). All control groups received an equal volume of vehicles. All drugs and chemicals were injected intraperitoneally (i.p.) at a volume of 10 mL/kg body weight unless otherwise indicated. The LDN-212320 and DHK injections were administered 24 h and 0.5 h prior to the behavioral experiments, respectively (Figure 1). The LDN-212320, DHK, and gabapentin doses were selected based on previous studies [41,42,43].

### 2.3. CFA-Induced Allodynia and Hyperalgesia

The CFA-induced allodynia and hyperalgesia, an established animal model for chronic inflammatory pain, was used as described previously [44,45]. Briefly, the left hind paw of each mouse was disinfected with 75% alcohol and injected intraplanar (i.pl.) with complete Freund’s adjuvant (1 mg/mL, 20 µL). The control animals were injected i.pl. with the same volume of vehicle into the left hind paw. All biochemical experiments were conducted 7 days after control (vehicle) or CFA i.pl. injection, when symptoms of persistent inflammatory pain were evident.

Tactile allodynia was performed as described previously [46] with minor modifications. Briefly, on days 1, 3, and 7 post i.pl. CFA injection, the mice were placed individually in a plastic cage (45 × 5 × 11 cm) with a wire mesh bottom, which allowed full access to the paws. In addition, behavioral acclimatization was allowed for 30 min. A 50% paw withdrawal threshold (50% PWT) against mechanical stimulation by von Frey filament (Stoelting, Inc., Wood Dale, IL, USA) to the plantar surface of each hind paw was measured using the up–down paradigm [47]. Based on preliminary studies that characterized the threshold stimulus in control animals, the innocuous 0.04 g (2.44) filament, representing 50% of the threshold force, was used to detect tactile allodynia. The filament was applied to the point of bending six times each to the dorsal surfaces of the left and right hind paws. Positive responses such as prolonged hind paw withdrawal followed by licking, biting, or scratching were recorded. Mice were tested 3 days before i.pl. injection of the CFA or vehicle to determine baseline thresholds, and then tested at 3 h, 1 day, 3 days and 7 days after i.pl. injection of the vehicle or CFA.

Thermal hyperalgesia was performed as described previously with minor modifications [46,48]. Briefly, the animals were handled twice a day for 3 days prior to the experimental procedures to habituate them to handle stress. The thermal hyperalgesia was measured by paw withdrawal latency from a hot plate using a plantar analgesia apparatus (IITC Life Science Inc., Woodland Hills, CA, USA). To measure the latency time, each mouse was individually placed on a hot plate maintained at 51 ± 0.5 °C in a Plexiglas chamber. The animals’ licking, flicking, or jumping was recorded as a positive response. The latency time for each mouse was calculated as a mean of three measurements with a 3 min interval between measurements. A cut-off time of 20 s was selected to prevent tissue damage. The mice were tested 3 days before i.pl. injection of the CFA or vehicle to determine the baseline thresholds, and then at 3 h, 1 day, 3 days, and 7 days after i.pl. injection of the vehicle or CFA.

The raw data from the CFA-induced allodynia and hyperalgesia were converted to the area under the curve (AUC). The AUC depicting the total paw withdrawal threshold versus time was computed using a trapezoidal calculation. The doses of LDN-212320, gabapentin, and DHK were used as described previously [41,42,43].

### 2.4. Western Blot Analysis

A Western blot analysis was performed as described previously [26,49]. Briefly, the mice were euthanized through rapid decapitation; their anterior cingulate cortex and hippocampi were dissected using mouse brain stereotaxic coordinates [50] and stored at −80 °C until the analysis. The hippocampus and ACC coordinates were based on the stereotaxic plates of the atlas of Franklin and Paxinos [50]; the anterior–posterior (AP) coordinates referred to the bregma, mediolateral (ML) coordinates to the midsagittal suture line, and ventral (DV) coordinates to the surface of the skull: CA1 (AP: −1.70 mm; ML: 1.17 mm and DV: 1.34 mm), DG (AP: −1.70 mm; ML: 0.70 mm and DV: 2.04 mm), and ACC (AP: 0.14 mm; ML: 0.25 mm and DV: 1.00 mm). The brain tissue was homogenized in modified RIPA buffer containing Dulbecco’s phosphate-buffered saline (PBS) (pH 7.4), 1% Igepal CA-630, 0.1% sodium dodecyl sulfate (SDS), protease inhibitor mix (cOmplete Mini Roche, Indianapolis, IN, USA), and phosphatase inhibitor (Catalog #: A32957, ThermoFisher Scientific, Waltham, MA, USA). The samples were centrifuged (16,000× *g*, 20 min at 4 °C) and the supernatant was collected and stored at −80 °C. The protein concentration was determined using a bicinchoninic acid assay (BCA) (Pierce, Rockford, IL, USA) using albumin as the standard. Equal amounts of protein (60 µg) were loaded onto 10% or 12.5% gels for SDS polyacrylamide gel electrophoresis. The separated proteins were transferred onto nitrocellulose membranes or polyvinylidene difluoride (PVDF) membranes at 60 V overnight at 4 °C. The membranes were blocked with 5% non-fat dry milk in Tris-buffered saline and 0.1% Tween-20 (TBST) for 1 h and were subsequently incubated overnight at 4 °C with primary antibodies for GLT-1 (1:1000, rabbit polyclonal, Santa Cruz Biotechnology, Dallas, TX, USA), CD11b (1:1000, rabbit polyclonal, Novus Biologicals, Centennial, CO, USA), Iba1 (1:500, goat polyclonal, Santa Cruz Biotechnology, Dallas, TX, USA), or β-actin (1:1000, rabbit polyclonal, Santa Cruz Biotechnology, Dallas, TX, USA). After incubation, the membranes were washed in TBST, followed by incubation with an appropriate horseradish-peroxide-conjugated secondary antibody and dilution in blocking buffer at a concentration of 1:5000. The bound antibodies were detected with ECL Prime reagent (Amersham, Buckinghamshire, UK), and the protein quantification was performed using a densitometric analysis.

### 2.5. Immunofluorescence Assay

The immunofluorescence assay was carried out as described previously [26] with minor modifications. Briefly, the mice were euthanized through rapid decapitation and their brains were removed and postfixed in 4% paraformaldehyde fixative overnight at room temperature. The brains were cryoprotected by immersion in 30% sucrose in 0.1 M PBS at 4 °C until the brains sank to the bottom. The brain tissues were embedded with Tissue- Tek OCT (Sakura Finetek USA Inc, Torrance, CA, USA) and sectioned into 15–20-μm-thick sections with a Leica CM1850 cryostat (Leica, Wetzlar, Germany). The sections were blocked with 5% normal goat serum in 0.3% Triton X-100 in 1× PBS for 1 h at room temperature then incubated overnight at 4 °C with anti-p38 (1:100, rabbit polyclonal, Cell signaling technology, Danvers, MA, USA) or CX43 (1:200, rabbit polyclonal, PhosphoSolutions, Aurora, CO, USA). After incubation, the sections were washed with PBS followed by incubation with Alexa Fluor 488 (AF488) conjugated secondary antibodies (1:200; ab 150077, Abcam, Cambridge, MA, USA) for 1 h at room temperature in a dark place. The slides were mounted with mounting medium containing 4′, 6′-diamidino-2-phenylindole (DAPI) for nuclear staining with anti-fade reagent (SouthernBiotech, Birmingham, AL, USA). The stained sections were then examined with an Olympus AX70 Olympus epifluorescence microscope attached to a DP70 Digital Camera (Tokyo, Japan). Image J software (v1.8.0, NIH, Bethesda, MD, USA) was used to quantify the expression of target protein bands using their integrated density.

### 2.6. IL-1β Estimation by ELISA

The IL-1β protein levels were determined using a mouse-specific ELISA kit (Invitrogen, Waltham, MA, USA). The mice were euthanized through rapid decapitation; their hippocampi and anterior cingulate cortex were harvested and stored at −80 °C until the analysis. The tissue samples were placed in sterile PBS containing a protease inhibitor cocktail (cOmplete Mini Roche, Indianapolis, IN, USA), homogenized, and centrifuged (11,000 rpm, 20 min, 4 °C), and the supernatant was assayed for IL-1β according to the manufacturer’s protocol. The protein concentrations of all samples were measured using a BCA protein assay kit (Pierce, Rockford, IL, USA) prior to the ELISA test, and equivalent amounts of proteins were used for the analysis. The cytokine levels are expressed as pg/mg of tissue.

### 2.7. Data Analyses

The data were analyzed by a two-way analysis of variance (ANOVA) followed by Tukey’s post hoc test to compare the behavioral measures between the experimental groups at different time points using GraphPad Prism 5.0 (GraphPad Inc., San Diego, CA, USA). The area under the curve (AUC) of the time course was analyzed using a one-way ANOVA followed by Tukey’s post hoc test. The biochemical studies were analyzed using a one-way ANOVA followed by Tukey’s post hoc test. The data from Western blot studies for GLT-1, CD11b, or Iba1/β-actin expression are presented as % of the control and the results are expressed as means ± S.E.M. The difference between treatments was considered significant at *p* < 0.05.

## 3. Results

### 3.1. Effects of LDN-212320 or Gabapentin on CFA-Induced Tactile Allodynia and Thermal Hyperalgesia

**Figure 2:**
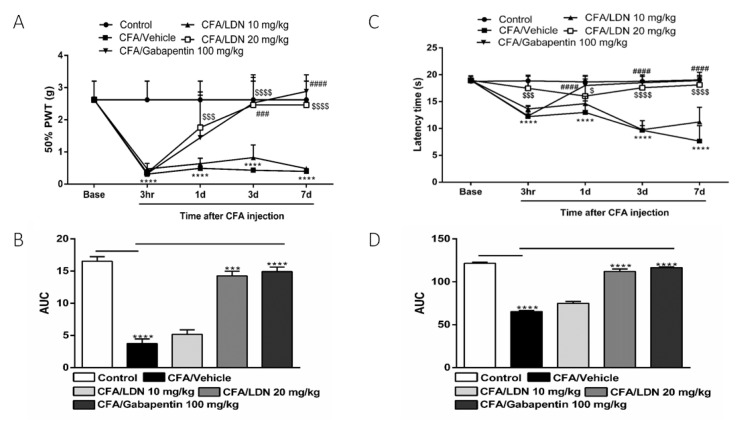
Effects of LDN-212320 or gabapentin on the 50% paw withdrawal threshold (50% PWT) during CFA-induced chronic inflammatory pain: (**A**) the 50% PWT was assessed using von Frey filaments 3 h, 1 day, 3 days, and 7 days after CFA (1 mg/mL, i.pl) administration; (**C**) effects of LDN-212320 or gabapentin on latency time on hotplate test (51 ± 0.5 °C) during CFA-induced chronic inflammatory pain, where the latency time on the hotplate was measured 3 h, 1 day, 3 days, and 7 days after CFA (1 mg/mL, i.pl) administration (**B**,**D**). The area under the curve (AUC) of the time course of the different behavioral tests was calculated by the trapezoidal rule. Control animals received an equal volume of vehicle (i.pl). Data are expressed as means ± S.E.M. (n = 4–6 mice/group). Note: *** *p* < 0.001, **** *p* < 0.0001, control (20 µL of vehicle, i.pl) vs. CFA/vehicle (1 mg/mL), ^$^ *p* < 0.05, ^$$$^ *p* < 0.001, or ^$$$$^ *p* < 0.0001, CFA/vehicle (1 mg/mL) vs. CFA/LDN-212320 (20 mg/kg) and ^###^ *p* < 0.001 or ^####^ *p* < 0.0001, CFA/vehicle (1 mg/mL) vs. CFA/gabapentin (100 mg/kg). AUC *** *p* < 0.001, **** *p* < 0.0001.

To evaluate the anti-allodynic effects of LDN-212320 or gabapentin on CFA-induced tactile allodynia, we assessed the development of allodynia at 3 h, 1 day, 3 days, and 7 days after unilateral hind paw intraplantar CFA injection. As shown in Figure 2A, the CFA (1 mg/mL, 20 µL) significantly (*p* < 0.0001) decreased the 50% paw withdrawal threshold at 3 h, 1 day, 3 days, and 7 days compared to the control, indicating the presence of tactile allodynia. Moreover, the pretreatment with LDN-212320 (20 mg/kg) or gabapentin significantly (*p* < 0.0001) increased the 50% paw withdrawal threshold. The two-way ANOVA revealed that the systemic administration of LDN-212320 (20 mg/kg) significantly attenuated the CFA-induced allodynia at 1 day, 3 days, and 7 days (F 4.80 = 36.89, *p* < 0.0001), indicating the anti-allodynic effects of LDN-212320 (20 mg/kg). Similarly, the pretreatment with gabapentin (100 mg/kg) significantly (*p* < 0.0001) increased the 50% paw withdrawal threshold. The two-way ANOVA revealed that the systemic administration of gabapentin (100 mg/kg) significantly attenuated the CFA-induced allodynia at 3 days and 7 days (F 4.20 = 24.53, *p* < 0.0001) but not at 1 day as observed with the LDN-212320 pretreatment (Figure 2A). Accordingly, the one way-ANOVA revealed that the overall effects (AUC) of LDN-212320 (20 mg/kg) or gabapentin (100 mg/kg) significantly (*p* < 0.0001) attenuated the CFA-induced allodynia (F 5.5 = 143.2, *p* < 0.0001) compared to the control injected with the vehicle, as shown in Figure 2B. Moreover, the pretreatment of LDN-212320 (10 mg/kg) did not significantly increase the 50% paw withdrawal threshold (Figure 2A, B). 

To determine the effects of LDN-212320 or gabapentin on CFA-induced thermal hyperalgesia, we evaluated the development of hyperalgesia at 3 h, 1 day, 3 days, and 7 days after intraplantar CFA injection. As shown in Figure 2C, the CFA (1 mg/mL, 20 µL) significantly (*p* < 0.0001) decreased the latency time on the hot plate as compared to the control, indicating a marked reduction in the response to the heat stimulus. Moreover, the pretreatment of LDN-212320 (20 mg/kg) significantly (*p* < 0.0001) increased the latency time on the hot plate test. The two-way ANOVA revealed that the systemic administration of LDN-212320 (20 mg/kg) significantly attenuated the CFA-induced thermal hyperalgesia at 3 h, 1 day, 3 days, and 7 days (F 4.16 = 37.84, *p* < 0.0001), indicating the anti-hyperalgesic effects of LDN-212320 (20 mg/kg) (Figure 2C). Similarly, the pretreatment of gabapentin (100 mg/kg) significantly (*p* < 0.0001) increased latency time on the hot plate test. The two-way ANOVA revealed that the systemic administration of gabapentin (100 mg/kg) significantly attenuated the CFA-induced thermal hyperalgesia at 1 day, 3 days, and 7 days (F 4.16 = 49.26, *p* < 0.0001) (Figure 2C). The one way-ANOVA revealed that LDN-212320 (20 mg/kg) or gabapentin (100 mg/kg) significantly (*p* < 0.0001) attenuated the CFA-induced thermal hyperalgesia (F 5.5 = 396.8, *p* < 0.0001) compared to the control injected with the vehicle, as shown in Figure 2D. However, the pretreatment of LDN-212320 (10 mg/kg) did not cause significantly increased latency times on the hot plate test (Figure 2C,D).

### 3.2. Effects of DHK on the Anti-Allodynic and Anti-Hyperalgesic Effects of LDN-212320

**Figure 3:**
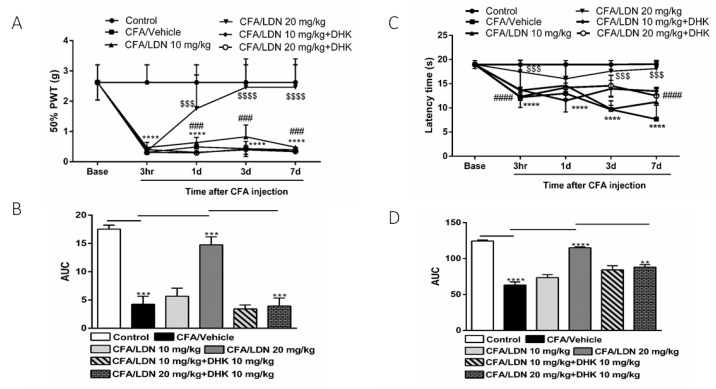
Effects of the astroglial GLT-1 antagonist, dihydrokainic acid (DHK), on LDN-212320-induced anti-allodynic and anti-hyperalgesic effects during CFA-induced chronic inflammatory pain: (**A**) the 50% paw withdrawal threshold (50% PWT) was assessed using von Frey filaments 3 h, 1 day, 3 days, and 7 days after CFA (1 mg/mL, i.pl) administration; (**C**) latency times on a hotplate test (51 ± 0.5 °C) were measured 3 h, 1 day, 3 days, and 7 days after CFA (1 mg/mL, i.pl) administration, while DHK (10 mg/kg) was injected 30 min before all behavioral experiments (**B**,**D**). The area under the curve (AUC) of the time course of the different behavioral tests was calculated using the trapezoidal rule. Control animals received an equal volume of vehicle (i.pl). Data are expressed as means ± S.E.M. (n = 4–6 mice/group). Note: **** *p* < 0.0001, control (20 µL of vehicle, i.pl) vs. CFA/vehicle (1 mg/mL), ^$$$^ *p* < 0.001 or ^$$$$^ *p* < 0.0001, CFA/vehicle (1 mg/mL) vs. CFA/LDN-212320 (20 mg/kg) and ^###^ *p* < 0.001 or ^####^ *p* < 0.0001, CFA/LDN-212320 (20 mg/kg) vs. CFA/LDN-212320 (20 mg/kg) plus DHK (10 mg/kg). AUC ** *p* < 0.01, *** *p* < 0.001, or **** *p* < 0.0001.

To examine if the anti-allodynic and anti-hyperalgesic effects of LDN-212320 are mediated by GLT-1 upregulation, we used a selective GLT-1 antagonist, dihydrokainic acid (DHK, 10 mg/kg). The pretreatment with DHK (10 mg/kg) significantly (*p* < 0.0001) reduced the 50% paw withdrawal threshold as compared to the LDN-212320 (20 mg/kg)-treated group (Figure 3A). Multiple comparisons of the means revealed that the administration of DHK (10 mg/kg) significantly attenuated the LDN-212320-induced anti-allodynic effects at 1 day, 3 days, and 7 days (F 4.96 = 96.11, *p* < 0.0001), confirming that the anti-allodynic effects of LDN-212320 were mediated by the GLT-1 transporter. The one way-ANOVA revealed that DHK (10 mg/kg) significantly (*p* < 0.001) attenuated the LDN-212320-induced anti-allodynic effects (F 5.6 = 51.51, *p* < 0.001), as shown in Figure 3B. In addition, the pretreatment with DHK (10 mg/kg) significantly (*p* < 0.0001) reduced the latency time on the hot plate test as compared to the LDN-212320 (20 mg/kg)-treated group (Figure 3C). Multiple comparisons of the means revealed that the administration of DHK (10 mg/kg) significantly attenuated the anti-hyperalgesic effects of LDN-212320 at 1 day, 3 days, and 7 days (F 4.96 = 48.61, *p* < 0.0001), confirming that the GLT-1 transporter mediated the anti-hyperalgesic effects of LDN-212320. Accordingly, the one way-ANOVA revealed that DHK (10 mg/kg) significantly (*p* < 0.01) attenuated the LDN-212320-induced anti-hyperalgesic effects (F 5.6 = 80.19, *p* < 0.01), as shown in Figure 3D.

### 3.3. Effects of LDN-212320 on Microglial Iba1 Expression in the Hippocampus and ACC

**Figure 4:**
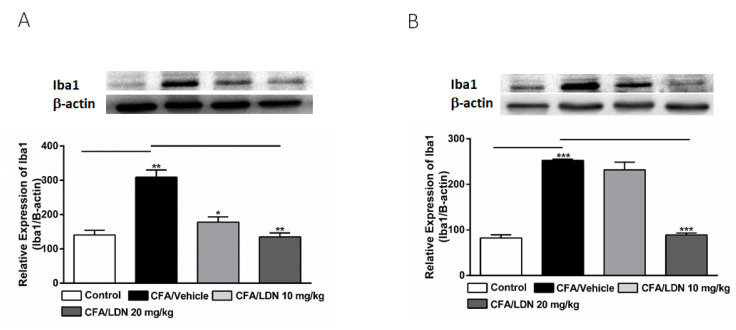
Effects of LDN-212320 on microglial Iba1 expression in the hippocampus (**A**) and anterior cingulate cortex (ACC) (**B**) on CFA-induced chronic inflammatory pain. Representative Western blot images for Iba1 expression from the hippocampus (**A**) and ACC (**B**) (top panel). CFA (i.pl) injection increased the Iba1 expression in the hippocampus (**A**) and ACC (**B**) compared to the control. Treatment of LDN-212320 (10 or 20 mg/kg) reversed the CFA-induced increased Iba1 expression in the hippocampus (**A**) or LDN-212320 (20 mg/kg) in the ACC (**B**). Control animals received an equal volume of vehicle (i.pl). Data are expressed as means ± S.E.M. (n = 4–6 mice/group). Note: * *p* < 0.05, ** *p* < 0.01, and *** *p* < 0.001.

To study the effects of LDN-212320 on microglial activation, the Iba1 protein expression in the hippocampus and ACC was quantified via an immunoblot analysis following CFA-induced chronic inflammatory pain. Interestingly, the intraplantar injection of CFA (1 mg/mL, 20 µL) into the hind paw significantly increased the Iba1 expression in the hippocampus (*p* < 0.01, Figure 4A) and ACC (*p* < 0.001, Figure 4B). Moreover, the one-way ANOVA showed a main effect of LDN-212320 (20 mg/kg) treatment on Iba1 expression in the hippocampus and ACC (*p* < 0.01, Figure 4A and *p* < 0.001 Figure 4B, respectively). Multiple comparisons of the means revealed that the administration of LDN-212320 (10 or 20 mg/kg) significantly decreased the Iba1 expression in the hippocampus (F 3.15 = 25.62, *p* < 0.01, Figure 4A) and at a higher dose (20 mg/kg) in the ACC (F 3.9 = 120.1, *p* < 0.0009, Figure 4B) compared to the CFA-injected group. However, the pretreatment with the lower dose of LDN-212320 (10 mg/kg) did not significantly decrease Iba1 expression in the ACC (Figure 4B).

### 3.4. Effects of LDN-212320 on Microglial CD11b Expression in the Hippocampus and ACC

**Figure 5:**
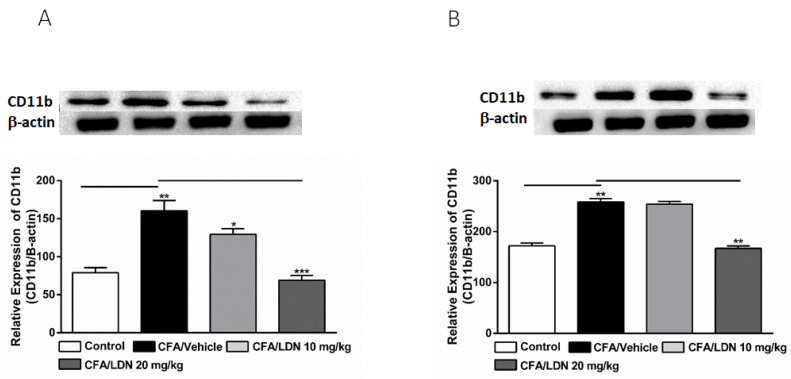
Effects of LDN-212320 on microglial CD11b expression in the hippocampus (**A**) and anterior cingulate cortex (ACC) (**B**) during CFA-induced chronic inflammatory pain. Representative Western blot images for CD11b expression from the hippocampus (**A**) and ACC (**B**) (top panel). CFA (i.pl) injection increased CD11b expression in the hippocampus (**A**) and ACC (**B**) compared to the control. Treatment of LDN-212320 (10 or 20 mg/kg) reversed the CFA-induced increased CD11b expression in the hippocampus (**A**) or LDN-212320 (20 mg/kg) in the ACC (**B**). Control animals received an equal volume of vehicle (i.pl). Data are expressed as means ± S.E.M. (n = 4–6 mice/group). Note: * *p* < 0.05, ** *p* < 0.01, and *** *p* < 0.001.

To evaluate the effects of LDN-212320 on microglial CD11b levels in the hippocampus and ACC, we assessed the CD11b expression in the hippocampus or ACC following CFA-induced chronic inflammatory pain. We found that CFA (i.pl.,1 mg/mL, 20 µL) injected into the hind paw significantly increased the CD11b expression in the hippocampus (*p* < 0.01, Figure 5A) and ACC (*p* < 0.01, Figure 5B). Moreover, the one-way ANOVA showed a main effect of the LDN-212320 (10 or 20 mg/kg) treatment on the CD11b expression in the hippocampus (*p* < 0.05, or *p* < 0.001, Figure 5A) and LDN-212320 (20 mg/kg) in the ACC (*p* < 0.01 5B). Multiple comparisons of the means revealed that the administration of LDN-212320 (10 or 20 mg/kg) significantly decreased the CD11b expression in the hippocampus (F 3.12 = 19.08, *p* < 0.0002, Figure 5A) and achieved the same at 20 mg/kg in the ACC (F 3.9 = 72.17, *p* < 0.0072, Figure 5B) compared to the CFA-injected group. The pretreatment of LDN-212320 (10 mg/kg) did not significantly decrease the CD11b expression in the ACC (Figure 5B).

### 3.5. Effects of LDN-212320 on Microglial p38 Immunoreactivity in the Hippocampus and ACC

**Figure 6:**
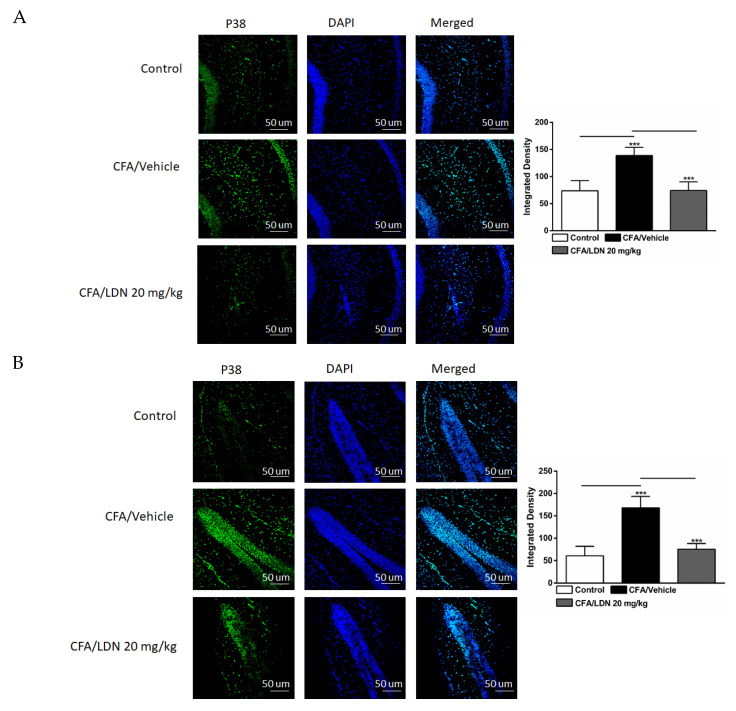

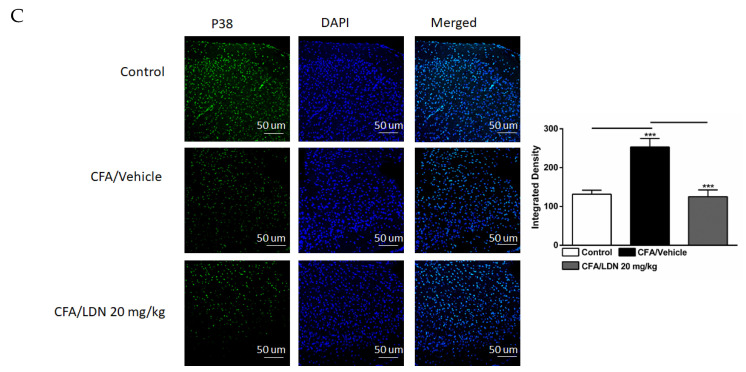
Effects of LDN-212320 on microglial p38 immunoreactivity in the CA1 and dentate gyrus (DG) of the hippocampus and anterior cingulate cortex (ACC) in CFA (1 mg/mL. i.pl)-induced chronic inflammatory pain. CFA (i.pl) injection increased the p38 immunoreactivity in the CA1 (**A**), DG (**B**), and ACC (**C**) compared to the control. LDN-212320 (20 mg/kg) treatment decreased the CFA-induced increased p38 immunoreactivity in the CA1 (**A**), DG (**B**), and ACC (**C**). Magnification 20×, scale bars, 50 µm. Representative images of immunofluorescence in (**A**–**C**) for the CA1 and DG of the hippocampus and ACC, respectively. Control animals received an equal volume of vehicle (i.pl). Data are expressed as means ± S.E.M. (n = 4–6 mice/group). Note: *** *p* < 0.001.

To determine the effects of LDN-212320 on p38 mitogen-activated protein kinase in the CA1 and dentate gyrus (DG) regions of the hippocampus and ACC during CFA-induced chronic inflammatory pain, we quantified the p38 immunoreactivity in these regions. A one-way ANOVA indicated that CFA (i.pl., 1 mg/mL, 20 µL) injected into the hind paw increased the p38 immunoreactivity significantly in the CA1 (*p* < 0.001), DG (*p* < 0.001), and ACC (*p* < 0.001) (Figure 6A–C, respectively) compared to the control. In addition, a post hoc test for multiple comparisons showed that LDN-212320 (20 mg/kg) reduced the p38 immunoreactivity significantly in the CA1 (*p* < 0.001), DG (*p* < 0.001), and ACC (*p*< 0.001) (Figure 6A–C, respectively). The pretreatment with LDN-212320 (20 mg/kg) significantly attenuated the CFA-induced increased p38 immunoreactivity in the CA1, DG, and ACC compared to the CFA-treated group (F 2.15 = 29.82, *p* < 0.0001; F 2.9 = 32.07, *p* < 0.0001; or F 2.9 = 22.10, *p* < 0.0003, respectively).

### 3.6. Effects of LDN-212320 on Astroglial GLT-1 Expression in the Hippocampus and ACC

**Figure 7:**
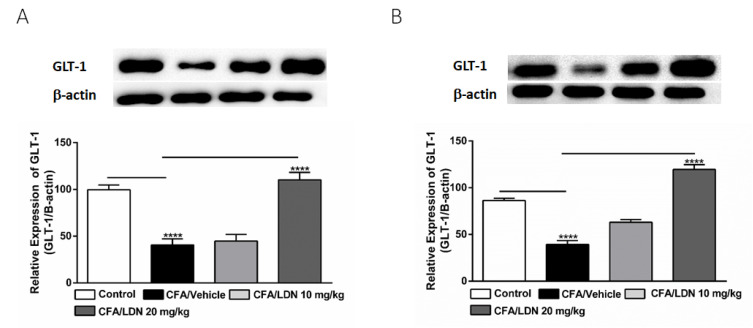
Effects of LDN-212320 on astroglial GLT-1 expression in the hippocampus (**A**) and anterior cingulate cortex (ACC) (**B**) during CFA-induced chronic inflammatory pain. Representative Western blot images for GLT-1 expression from the hippocampus (**A**) and ACC (**B**) (top panel). CFA (i.pl) injection decreased the GLT-1 expression in the hippocampus (**A**) and ACC (**B**) compared to the control. Treatment with LDN-212320 (20 mg/kg) reversed the CFA-induced decreased GLT-1 expression in the hippocampus (**A**) and ACC (**B**). Control animals received an equal volume of vehicle (i.pl). Data are expressed as means ± S.E.M. (n = 4–6 mice/group). Note: **** *p* < 0.0001.

To investigate the effects of LDN-212320 on the astroglial GLT-1 levels in the hippocampus and ACC, we quantified the GLT-1 expression in the hippocampus or ACC following CFA-induced chronic inflammatory pain. Interestingly, CFA (i.pl., 1 mg/mL, 20 µL) injected into the hind paw significantly decreased the GLT-1 expression in both the hippocampus and ACC (*p* < 0.0001, Figure 7A,B, respectively). Moreover, the one-way ANOVA showed a main effect of LDN-212320 (20 mg/kg) treatment on the GLT-1 expression in the hippocampus and ACC (*p* < 0.0001, Figure 7A,B, respectively). Multiple comparisons of the means revealed that the administration of LDN-212320 (20 mg/kg) significantly increased the GLT-1 expression in the hippocampus (F 3.26 = 29.10, *p* < 0.0001, Figure 7A) and ACC (F 3.28 = 81.14, *p* < 0.0001, Figure 7B) compared to the CFA-injected group. Again, the pretreatment with the lower dose of LDN-212320 (10 mg/kg) had no effect and did not significantly increase the GLT-1 expression in the hippocampus or ACC (Figure 7A,B, respectively).

### 3.7. Effects of LDN-212320 on Astroglial CX43 Immunoreactivity in the Hippocampus and ACC

**Figure 8:**
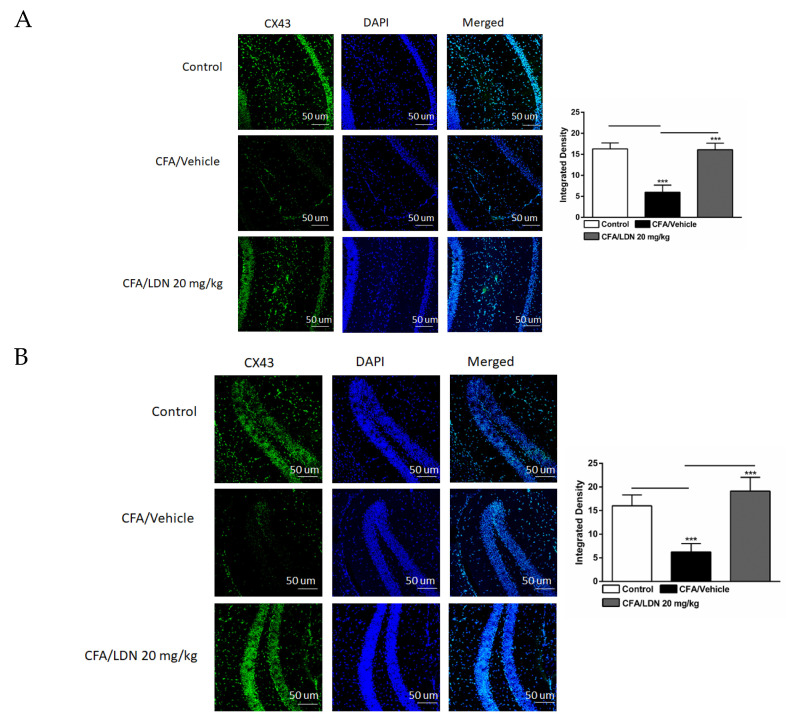

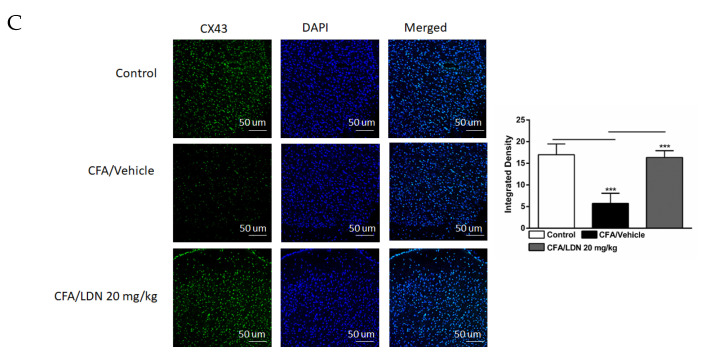
Effects of LDN-212320 on astroglial CX43 immunoreactivity in the CA1 and dentate gyrus (DG) of the hippocampus and anterior cingulate cortex (ACC) in CFA (1 mg/mL. i.pl)-induced chronic inflammatory pain. CFA (i.pl) injection decreased the CX43 immunoreactivity in the CA1 (**A**), DG (**B**), and ACC (**C**) compared to the control. LDN-212320 (20 mg/kg) treatment increased the CFA-induced decreased CX43 immunoreactivity in the CA1 (**A**), DG (**B**), and ACC (**C**). Magnification 20×, scale bars, 50 μm. Representative images of immunofluorescence in (**A**–**C**) for the CA1 and DG of the hippocampus and ACC, respectively. Control animals received an equal volume of vehicle (i.pl). Data are expressed as means ± S.E.M. (n = 4–6 mice/group). Note: *** *p* < 0.001.

To assess the effects of LDN-212320 on astroglial CX43 in the CA1 and dentate gyrus (DG) regions of the hippocampus and ACC during CFA-induced chronic inflammatory pain, we examined the CX43 immunoreactivity in these regions. The one-way ANOVA indicated that the intraplantar injection of CFA (1 mg/mL, 20 µL) into the hind paw decreased the CX43 immunoreactivity significantly in the CA1 (*p* < 0.001), DG (*p* < 0.001), and ACC (*p* < 0.001) (Figure 8A–C, respectively) compared to the control. In addition, the one-way ANOVA showed that LDN-212320 (20 mg/kg) prevented CFA-mediated reduced CX43 immunoreactivity in the CA1 (*p* < 0.001), DG (*p* < 0.001), and ACC (*p* < 0.001) (Figure 8A–C, respectively). The pretreatment with LDN-212320 (20 mg/kg) significantly reduced the CFA-induced decreased CX43 immunoreactivity in the CA1, DG, and ACC compared to the CFA-treated group (F 2.15 = 86.27, *p* < 0.0001; F 2.12 = 39.91, *p* < 0.0001; F 2.9 = 34.22, *p* < 0.0001, respectively).

### 3.8. Effects of LDN-212320 on IL-1β Level in the Hippocampus and ACC

**Figure 9:**
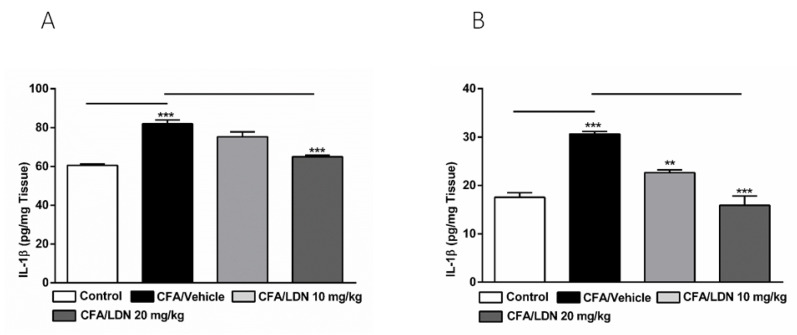
Effects of LDN-212320 on IL-1β levels in the hippocampus and anterior cingulate cortex (ACC). CFA (i.pl) injection increased the IL-1β levels in the hippocampus (**A**) and ACC (**B**) compared to the control. Treatment with LDN-212320 (20 mg/kg) decreased the IL-1β level in the hippocampus (**A**) or (10 or 20 mg/kg) ACC (**B**). Control animals received an equal volume of vehicle (i.pl). Data are expressed as means ± S.E.M. (n = 4–6 mice/group). Note: ** *p* < 0.01, *** *p* < 0.001.

To determine the effects of LDN-212320 on proinflammatory mediators produced by glial cells in the hippocampus and ACC, we examined the IL-1β protein expression in these regions following CFA-induced chronic inflammatory pain. The one-way ANOVA indicated that the intraplantar injection of CFA (1 mg/mL, 20 µL) into the hind paw increased the IL-1β levels significantly (*p* < 0.001) in the hippocampus and ACC (Figure 9A,B, respectively). Moreover, the one-way ANOVA revealed that the LDN-212320 pretreatment had a significant effect (*p* < 0.001) on the IL-1β levels in the hippocampus and ACC. A post hoc test for multiple comparisons revealed that the higher dose of LDN-212320 (20 mg/kg) significantly attenuated the CFA-induced increased IL-1β levels in the hippocampus (F 3.12 = 33.82, *p* < 0.0001), as did both 10 and 20 mg/kg in the ACC (F 3.12 = 33.10, *p* < 0.0001) (Figure 9A,B, respectively).

## 4. Discussion

In the present study, the major findings are that the GLT1 activator LDN-212320 significantly attenuated allodynia and hyperalgesia associated with a CFA-induced model of chronic inflammatory pain. We found that the anti-allodynic and anti-hyperalgesic effects of LDN-212320 were prevented by pretreatment with DHK, a GLT-1 antagonist. In addition, LDN-212320 significantly reversed CFA-induced increases in microglial activation, decreased astroglial GLT-1 and CX43 expression, and increased the inflammatory mediator IL-1β’s levels in the hippocampus and ACC. Thus, the present study reveals novel mechanisms of LDN-212320 in the hippocampus and ACC associated with pain modulation in chronic inflammatory pain. To the best of our knowledge, we show here for the first time the cellular mechanism associated with GLT-1 modulation in chronic inflammatory pain induced by CFA.

Our study indicated that LDN-212320 inhibits microglial activation associated with chronic inflammatory pain induced by CFA. In accordance with these observations, previous reports found that suppressing microglial activation delayed the development of allodynia in a chronic pain model [6]. In addition, systemic treatment with minocycline, a microglial inhibitor, attenuated hyperalgesia, and allodynia in a rat model of neuropathic pain [11]. Importantly, we have demonstrated that intraplantar CFA administration increased the expression of the microglial markers Iba1 and CD11b and microglial-induced proinflammatory cytokine production in the hippocampus and ACC. These findings are consistent with previous studies showing that peripheral inflammation induces microglial activation and proinflammatory cytokine release in forebrain regions [51,52]. Previous reports also indicated that microglial activation plays an important role in pain facilitation induced by peripheral tissue injury [53,54,55]. It is noteworthy to mention that pharmacological approaches targeting microglial activation prevented microglial responses and subsequently attenuated chronic pain associated with peripheral tissue injury [56,57]. In fact, microglial activation induces a range of pronociceptive molecules that may potentiate pain transmission [58,59]. For example, p38 is predominantly expressed in the spinal microglia after peripheral nerve injury [60,61]. In addition, the activation of microglial cells following peripheral nerve injury facilitates persistent pain via p38 and IL-1β mediation [62,63]. It is worth mentioning that increased glutamate activity may induce microglial p38 activation to promote injury [64,65], which is in line with our observations. Moreover, our findings demonstrate that p38 upregulation is consistent with the activation of microglia within the hippocampus and ACC. This notion is also supported by previous reports regarding the p38-dependent activation of microglia and associated pain facilitation [8,66].

Consistent with our previous report on formalin-induced nociceptive pain [26], we have demonstrated that GLT-1 modulation inhibited microglial activation and reduced CFA-induced hyperalgesia and allodynia. These results are also consistent with previous findings showing that systemic treatment with ceftriaxone, a known GLT-1 activator, protected rats against cerebral ischemic injury by preventing microglial activation in the striatum [67]. Moreover, previous studies have shown that GLT-1 modulation prevents chronic pain [68,69]. For example, systemic treatment with ceftriaxone attenuated allodynia and hyperalgesia in animal models of chronic pain [37,70]. Furthermore, ceftriaxone treatment significantly reversed GLT-1 downregulation in a chronic pain model [69,71], which is consistent with our findings.

In this study, we found that astroglial CX43 is downregulated in the hippocampus and ACC following peripheral tissue injury. This is consistent with a recent report suggesting that decreased astroglial CX43 expression might facilitate pain transmission by enhancing algesic targets following nerve injury [72]. Interestingly, increasing spinal astroglial CX43 expression to normal levels inhibited mechanical hypersensitivity during chronic pain [39]. Furthermore, astroglial CX43 was shown to play a major role as a mediator of injury-dependent downregulation of GLT-1 in the hippocampus [73,74]. These observations support the notion that reduced GLT-1 expression could be partially attributed to astroglial CX43 downregulation following peripheral tissue injury. These results, in addition to our findings provide strong evidence that GLT-1 modulation by LDN-212320 in the hippocampus and ACC is important in attenuating chronic inflammatory pain.

Previous studies reported that increased glutamate accumulation activates glutamate ionotropic receptors leading to increased IL-1β synthesis and expression in microglial cells in the rat brain [75], supporting the notion that increased glutamate may be in part responsible for microglial activation in our model. Indeed, it was found that the application of glutamate in the rodent cerebral cortex sensitized microglial cells to produce inflammatory mediators [76], giving functional relevance to our observations. Importantly, it was shown that IL-1β significantly influenced the glutamatergic release in the CNS following peripheral tissue injury [77,78,79]. For example, IL-1β was found to increase the physiological function of the glutamate N-methyl-D-aspartate (NMDA) receptor by increasing the frequency of NMDA receptor channel opening [80] and directly enhancing the activity of hippocampal neuronal NMDA receptors [81]. In addition, IL-1β was shown to facilitate inflammatory pain by enhancing the phosphorylation of NMDA receptors [77]. Several studies have suggested that IL-1β might modulate presynaptic glutamatergic release via signaling pathways involving increased Ca^2+^ influx in the hippocampus [82,83,84]. Moreover, IL-1β was shown to decrease the expression of the astroglial GLT-1 leading to impaired glutamate transport [85]. Consistent with these reports, we have shown that the CFA-induced increase in IL-1β release could decrease the GLT-1 expression in the hippocampus and ACC, and this downregulation was reversed by LDN-212320. It is important to note that in the present study we focused on IL-1β mainly due to its critical involvement in the glutamatergic system, as noted previously, and for its role as a key mediator of neuroinflammatory responses. However, future studies are important to determine the effects of other proinflammatory cytokines on glutamatergic neurotransmission in chronic inflammatory pain.

It is well documented that glutamate transporters are also expressed in peripheral tissues [86]. Given this, LDN-212320 might have additional effects by targeting glutamate transporters expressed in these tissues. However, we have shown that the systemic treatment of LDN-212320 does not produce any noticeable change to the expression of peripheral inflammation markers [26], suggesting an insignificant role. However, it is possible that acute treatment in a formalin-induced nociceptive model could limit any observable effects of LDN-212320 in peripheral tissues. Therefore, future studies are necessary to examine the effects of LDN-212320 on the peripheral glutamate transporter system.

It is widely believed that the hippocampus and ACC play an important role in pain perception and modulation [87,88]. Pertinent to this study, peripheral nerve injury induces robust glutamate release in the hippocampus and ACC [89,90]. Furthermore, previous studies demonstrated that increased glutamatergic neurotransmission modulates microglial activation directly through glutamate receptors or indirectly via extracellular adenosine triphosphate signaling in response to glutamate release [91,92]. More importantly, increased extracellular glutamate release following nerve injury stimulates glutamate receptors, which in turn activate microglia to induce the release of cytokines such as IL-1β in the hippocampus [93], which could be relevant to peripheral nerve injury. Similarly, a previous study has shown that accumulated glutamate leads to microglial activation through the release of proinflammatory mediators [92]. It is of interest to state that microglia–neuron–astroglia crosstalk is evident in most peripheral injury models [4]. Importantly, it was found that microglial activation could modulate glutamate neurotransmission in the CNS following central insults [94]. For example, CX3C chemokine ligand 1 (CX3C1), acting on microglia, was shown to indirectly modify GLT-1 expression on astroglia via the release of soluble factors such as the adenosine A1 receptor [95]. Most interestingly, it was found that gabapentin reduces microglial activation through the blockage of CX3C1 signaling following CFA-induced peripheral inflammation [96], suggesting the crucial involvement of GLT-1 and CX3C1 in the crosstalk between microglia and astroglia. Furthermore, several studies support the notion that GLT-1 activity is greatly involved in glutamate receptor activation [97]. For instance, GLT-1 upregulation is considered the limiting step for the induction of long-term depression in the hippocampal synapse by modulating metabotropic glutamate receptor activation [98]. In accordance with these reports and from our observations, we propose that GLT-1 modulation controls microglial activation through mediators or the direct modulation of the glutamatergic system in microglial cells. Moreover, a previous report has shown that gabapentin decreased microglial activation in rodents with chronic pain [99]. Interestingly, other reports showed that reduced gabapentin effectiveness was associated with decreased GLT-1 expression [41], suggesting that gabapentin’s effect is GLT-1-dependent, which is similar to our findings with LDN-212320. However, future studies are required to confirm this notion in the CFA-induced chronic inflammatory pain model.

Previous evidence suggested that the excessive accumulation of glutamate may be responsible for central sensitization in chronic pain [100,101]. Glutamate accumulation may occur due to increased presynaptic release through increased neuronal activity [102,103] or impaired glutamate uptake caused by GLT-1 downregulation during peripheral tissue injury [26,104]. It is worth mentioning that the glutamate transporter competes with glutamate receptors for glutamate binding [97], suggesting that glutamate transporters critically modulate glutamate receptor activity and function. In accordance with this, we have shown that the removal of glutamate from the synapses through GLT-1 activation prevents microglial activation. Similarly, earlier data reported that the systemic administration of MK-801, an NMDA receptor antagonist, prevents microglial activation in the hippocampus [105,106,107].

We explicitly observed that glutamate transporter modulation in the hippocampus and ACC prevented microglial activation and extended our previous findings with LDN-212320 on formalin-induced nociceptive behavior in mice [26]. Our data demonstrate that LDN-212320 significantly attenuated microglial activation associated with CFA-induced chronic inflammatory pain, likely through the regulation of GLT-1 in the hippocampus and ACC. However, additional studies are necessary to confirm these observations by using site-specific intracranial injections of LDN-212320 in these brain regions. Future studies are warranted to elucidate the role of astroglial GLT-1 modulation using site-specific routes such as intracranial or intranasal administration. Intranasal administration can be preferred since it influences drug delivery to the brain. Additional studies should focus on lipid-based nanoformulations for an improved pharmacokinetic profile.

## 5. Conclusions

Our novel findings provide strong evidence that LDN-212320 prevents CFA-induced allodynia and hyperalgesia. This effect is likely mediated by reversing CFA-induced microglial activation and upregulating astroglial GLT-1 and CX43 expression in the hippocampus and ACC in a mouse model of inflammatory pain. Together, these findings give compelling support to the idea that LDN-212320 could be developed as a novel therapeutic drug candidate for chronic inflammatory pain.

It is noteworthy to mention that we have studied the effects of the astroglial GLT-1 modulation in the hippocampus and ACC in male mice. However, astroglial GLT-1 regulation in female mice is critical in acute and chronic inflammatory pain. Therefore, future studies are needed to examine the astroglial GLT-1 modulation in female mice for acute and chronic inflammatory pain.

## Data Availability

All data included in this study are available upon request from the corresponding authors.
